# Precision Prevention through Social Media: Report of Four Cases

**DOI:** 10.1055/s-0044-1800718

**Published:** 2025-04-08

**Authors:** Elia Gabarron, Guillermo Lopez-Campos, Shauna Davies, Taridzo Chomutare, Iris Thiele Isip Tan, Carolyn Petersen

**Affiliations:** 1 Department of Education, ICT and Learning, Østfold University College, Halden, Norway; 2 Norwegian Centre for E-health Research, University Hospital of North Norway, Tromsø, Norway; 3 Queen's University Belfast, Belfast, United Kingdom; 4 Faculty of Nursing, University of Regina, Canada; 5 Department of Computer Science, Faculty of Science and Technology, UiT The Arctic University of Norway, Tromsø, Norway; 6 Medical Informatics Unit, College of Medicine, University of the Philippines Manila, Philippines; 7 Department of Artificial Intelligence and Informatics, Mayo Clinic, Rochester, Minnesota, United States

**Keywords:** Social media, Prevention, Precision medicine, Internet-based intervention

## Abstract

**Background** : Precision prevention involves using biological, behavioral, socioeconomic, and epidemiological data to improve health for a particular individual or group. With almost 63% of the global population using social media, these platforms show promise to deliver tailored messaging and personalized interventions to individuals.

**Objectives** : To describe the personalization elements and behavior components used in a sample of precision prevention interventions delivered through social media.

**Methods** : To identify examples of cases, a search was done on clinicaltrials.gov, searching for ‘other terms: prevention’ + ‘Intervention/Treatment: social media intervention’ + ‘study results: With results. The selected cases were described, personalization elements reported, and their adopted intervention components were coded according to the Behavior Change Wheel (BCW) framework.

**Results** : A total of four cases employing personalization in their interventions were identified. Three of these cases targeted women's health. The intervention period varied from two to eight months, with participant commitment ranging from active involvement on five out of seven days to monthly participation. The BCW interventions of persuasion and incentivization, were most frequently utilized, while education and coercion were used sparingly in the selected cases. Notably, none of the four cases reported the use of training, restrictions, or modeling.

**Conclusions** : Social media has the potential to serve as a tool for digital phenotyping and contribute to the advancement of precision prevention. Challenges include the social media platform set-up and ensuring all ethical considerations are met.

## 1. Introduction

Despite being a relatively new technology, social media has quickly become one of the most popular and widely embraced forms of technology. By the end of 2023, there were almost 5 billion individuals, or roughly 63% of the global population, using social media [ 1 ]. The popularity and potential for engaging and communicating with individuals make social media a valuable tool for delivering and supporting health interventions [ 2 3 4 ].

In recent years, there has been a notable increase in the number of health research studies implementing interventions through social networks or utilizing social media to support other types of interventions. Several reviews that specifically summarize studies on social media interventions for health purposes are already published [ 5 6 7 8 9 10 11 12 ]. Many of these social media interventions have followed a “one-size-fits-all” approach that might not be beneficial for everyone. However, interventions delivered through social media can adopt tailored strategies, enabling more effective and personalized health promotion efforts and therefore contributing to the concept of precision prevention [ 13 ]. This concept, borrowed from preventive medicine, involves using biological, behavioral, socioeconomic, and epidemiological data to address the challenges posed by complex diseases and health disparities. The ultimate goal of precision prevention is to decrease disease incidence and mortality in a particular individual or group [ 14 , 15 ]. In the case of social media, for example, these channels could be utilized in precision prevention by leveraging targeted communication strategies, tailored messaging, and personalized interventions based on individual characteristics, behaviors, or preferences.

The Behavior Change Wheel (BCW) framework [ 16 ] is a toolkit that can facilitate the design of precision prevention interventions to be delivered through social media interventions by providing a systematic and comprehensive approach. The model encompasses three layers: capability, opportunity, motivation-behavior (COM-B); the policy categories; and the nine intervention components that allow for tailored strategies: Education (Increasing knowledge or understanding); Persuasion (Using communication to induce positive or negative feelings or stimulate action); Incentivization (Creating expectation of reward); Coercion (Creating expectation of punishment or cost); Training (Imparting skills); Restriction (Using rules to reduce the opportunity to engage in the target behavior or to increase the target behavior by reducing the opportunity to engage in competing behaviors); Environmental restructuring (Changing the physical or social context); Modelling (Providing an example for people to aspire to or imitate); Enabling (Increasing means/reducing barriers to increase capability or opportunity) [ 16 ]. Identifying and understanding the personalization elements and the behavioral components employed in real cases of prevention interventions delivered through social media, and the ways in which these channels are used could help improve behavior change interventions.

The objective of this paper is to describe the personalization elements and behavioral components employed in a sample of precision prevention interventions delivered through social media.

## 2. Methods

To identify examples of projects that use social media for delivering a personalized intervention for preventing disease/reducing morbidity, a search was performed on clinicaltrials.gov for ‚other terms: prevention‘ + ‚Intervention/Treatment: social media intervention‘ + ‚study results: With results‘. All the identified records were checked one by one. The selected cases were narrowed down to those projects that explicitly indicated the intervention was personalized. The search was done by EG, and the selected cases were verified by CP and GLC. To gather maximum information about the cases, we manually searched for additional publications related to the selected registries. CP and GLP described the selected cases and reported the personalization elements. EG coded the intervention components explicitly reported in these interventions according to the Behaviour Change Wheel (BCW) framework [ 16 ].

## 3. Results

The search on clinicaltrials.gov resulted in 16 records. After checking each of the records one by one, a total of four cases were identified that specifically referred to the use of personalization in their interventions [ 17 18 19 20 ]. Studies in 3 of the cases were conducted in the USA [ 17 , 18 , 20 ] while 1 study was conducted in Taiwan [ 19 ]. These interventions lasted between eight weeks and eight months.

### 3.1. Description of the cases

Summarized information on the four cases is presented in Table 1 . The table includes a description of the problem, the intervention, and results and impact. Three of the cases are related to women's health, specifically, reducing alcohol dependence among minority women (Case 1) [ 18 , 21 , 22 ], weight loss among women who recently gave birth (Case 2) [ 20 , 23 , 24 ], and physical exercise among older hypertensive women (Case 3) [ 19 , 25 ]. Two of the cases (Case 3 & 4) use personalized social media interventions to tackle risky behaviors such as alcohol dependency [ 17 , 19 , 25 ].

**Table 1.** Description of the four cases.

	Problem	Intervention	Results and impact
Case 1 [ 18 , 21 , 22 ]	Alcohol consumption and stigma-coping behaviors in sexual minority women	Social media: LexParlay, a social media online game, to deliver a personalized prevention intervention to reduce alcohol consumption Personalization element: culturally tailored tool and Personalized Normative Feedback (PNF) Intervention duration: 8 months n= 2,667	The effectiveness of alcohol PNF was moderated in recently stigma-exposed individuals. The use of culturally adapted PNF interventions might increase the effectiveness of PNF interventions in stigmatized populations. This study highlights the importance of stratifying target populations and tailoring the intervention to these subgroups of participants can increase the effectiveness of the intervention.
Case 2 [ 20 , 23 , 24 ]	Post-partum weight retention	Social media: Facebook, to deliver evidence-based weight loss programming to post-partum women Personalization element: individualized calorie and physical activity goals Intervention duration: 6 months n=62	The weight loss was significantly higher for those in the traditional in-person intervention; however, due to COVID-19, the final sessions were conducted online with self-reported weights, potentially introducing inaccuracies. This study demonstrates the value of face-to-face communication in effecting lifestyle change, i.e. weight loss. Facebook was not an effective substitute for in-person coaching sessions.
Case 3 [ 19 , 25 ]	Effect of exercise on cognitive function of older hypertensive women	Social media: Line, to study the effect of individualized education on memory, brain-derived neurotrophic factor (BDNF) levels, and subjective cognitive complaints in older hypertensive women Personalization element: personalized education session on how to perform the walking exercise Intervention duration: 24 weeks n= 58	Significant improvement in the delayed recall tasks of memory scores, however there were not significant differences for the total recall task of the memory scores, the subjective cognitive complaints nor in the concentration of BDNF. Despite no observed differences in BDNF levels or subjective cognitive impairment, the intervention, based on individualized education and counseling using telephone and social media, positively impacted memory function.
Case 4 [ 17 ]	Risky alcohol consumption	Social media: Snapchat, to prevent risky behavior and promote wellness. Personalization element: motivational interviewing methods delivered via private health coaching on social media Intervention duration: 8 weeks n= 94	In preliminary analysis phase (only demographics of included participants are reported).

In terms of the sample sizes, Case 1 had the most participants (n= 2,667) [ 18 , 21 , 22 ], while the other cases had a total of 214 participants combined. The majority of cases lasted at least six months, with the exception of Case 4 which had a duration of two months. In each of the cases, the interventions required different levels of commitment from the participants. For example, in Case 3, participants needed to dedicate 30 minutes per day, 5 days a week for the intervention [ 19 , 25 ], and in Case 1, participants only needed to engage in one round of gameplay monthly [ 18 , 21 , 22 ].

While the results from Case 4 are not yet published, the other three cases demonstrate varying effectiveness of personalized social media (Snapchat) as an intervention [ 17 ]. The results from Case 1 support stratification and targeting the identified subgroups with personalized interventions for reducing alcohol dependency among minority women [ 18 , 21 , 22 ]. Case 2, on the other hand, found that personalized social media (Facebook) was less effective than face-to-face interventions for postpartum weight-loss [ 20 , 23 , 24 ]. In Case 3, the results were mixed, showing greater improvement only for delayed recall in the aerobic walking group, compared to the control [ 19 , 25 ]. It is important to note, however, that the intervention in Case 3 has many components, and social media was used only for counseling [ 19 , 25 ].

### 3.2. Behavior components used in the personalized interventions

All cases used multiple BCW intervention components, except for Case 3, which reported only employing persuasion (see Table 2 ). Case 4 used the most number of components, namely persuasion, incentivization, education, coercion, enablement, and environmental restructuring [ 17 ]. While the social media intervention in Case 3 was offered only to those with mobile internet access as part of follow-up and feedback, social media messaging was the main intervention in Case 4, hence more behavior change components were evident [ 17 ].

**Table 2.** Examples of intervention functions adopted in each of the reviewed cases.

Used BCW Intervention components [ 16 ]	Case 1	Case 2	Case 3	Case 4
Persuasion (stimulate action)	«Personalized normative feedback on sexual identity and age specific descriptive drinking and stigma-coping norms are delivered to players within the context of an online competition designed to challenge sexual minority women stereotypes and increase visibility» [ 18 ]	«The weight loss counselor will facilitate discussions about weekly topics by engaging participants in problem solving, assisting them in setting SMART goals (...) providing constructive feedback, sharing resources, providing positive reinforcement, and answering questions» [ 23 ]	« (…) a 60-minute face-to-face individualized education session on how to perform 30 minutes of walking exercise five times a week, an intervention booklet, and follow-up counseling by telephone and social media every two weeks» [ 19 , 25 ]	«A study team member will reach out to you via your preferred method of contact. (...) The SnappyHour team will deliver health information focused on increasing well-being and reducing risky behaviors.» [ 17 ]
Incentivization (expectations of reward)	«You can earn points in each round by guessing and betting on the attitudes and behaviors of other LBQ women in your age-group and then reporting on your own attitudes & behaviors. In each round: a variable cash prize ranging from $50 to $500 will be awarded to the player who scores the most points that round.» [ 18 ]	«Participants will receive a $20 gift card after completing the baseline assessment (visit and survey) and a $40 gift card after completing each follow-up study assessment (6 months and 12 months) to compensate them for their time, for a total of $100.» [ 20 ]	Not reported	«You can earn up to $110 in electronic gift cards and your participation in the study will end when you complete your 4-month follow-up survey» [ 17 ]
Education (increase knowledge)	Not reported	«to cover the intervention content in that intervention module of the DPP lifestyle intervention through posts that provide information or resources, solicit sharing of thoughts or experiences or challenges related to the topic of the week» [ 23 ]	Not reported	«Participants randomized to the intervention will be initially provided with a link to the NIAAA's guide Rethinking Drinking website, a list of national resources located on our study website, and the study's Snapchat user safety agreement.» [ 17 ]
Coercion (expectations of punishment)	Not reported	«we included stock images of women with larger bodies with a variety of skin tones, racial or ethnic phenotypes, and family configurations» [ 24 ]	Not reported	«Intervention theme “Staying healthy”: Address physical/health motives (e.g., sleep, pain), long-term health impacts, avoiding risky behaviors, mental health» [ 17 ]
Enablement (Increase capability)	Not reported	Not reported	Not reported	«Intervention theme “Getting support”: Explore benefits of change, planning, and cognitive/behavioral strategies for making changes if desired» [ 17 ]
Environmental restructuring (change in physical environment)	Not reported	Not reported	Not reported	«Intervention theme “Staying out of trouble”: Discuss risk perceptions, protective behavioral strategies (e.g., avoid drunk driving), planning ahead (e.g., safe rides)» [ 17 ]
Training (impart skills)	Not reported	Not reported	Not reported	Not reported
Restrictions (reduce opportunity to engage in behavior)	Not reported	Not reported	Not reported	Not reported
Modelling (examples to imitate)	Not reported	Not reported	Not reported	Not reported
Total reported functions	2	4	1	6

Persuasion was the most commonly used intervention component and it was used in all four cases. This ranged from messaging by peer coaches (Case 4) [ 17 ], follow-up counseling via social media (Case 3) [ 19 , 25 ], to providing personalized normative feedback (Case 1) [ 18 ], providing positive reinforcement, and answering questions (Case 2) [ 23 ].

The next most common intervention component was incentivization, which was used in three of the cases. Economic incentives offered were either cash prizes or gift cards, ranging in value from $20 to $500. These incentives were earned according to a participant's performance in the game (Case 1) [ 18 ] or by the level of participation or completion of milestones (Cases 2 and 4) [ 17 , 20 ]. Earning points was also offered as incentive in the game (Case 1) [ 18 ].

Education was provided either through social media posts containing information or resources (Case 2) [ 23 ] or as website links (Case 4) [ 17 ]. Coercion took the form of stock images of women with large bodies in the training materials of a trial on postpartum weight loss (Case 2) [ 24 ] and the inclusion of social media messages on long-term health impacts and effects on mental health of risky drinking (Case 4) [ 17 ].

Case 4, which included the most BCW intervention components in its intervention, used enablement and environmental restructuring [ 17 ]. To reduce risky drinking, social media messages included planning, cognitive/behavioral strategies for making changes (enablement), and protective behavioral strategies like avoiding drunk driving and planning for safe driving (environmental restructuring).

None of the four cases report the use of the following BCW intervention components: training, restrictions, and modeling.

## 4. Discussion

### 4.1. Summary of findings

Rooted in the concept of precision medicine, precision prevention explores how the information-rich environment can be used to tailor specific preventative interventions. This approach customizes specific preventive measures based on detailed knowledge about the target individuals and populations and has been applied in different areas [ 13 14 15 ]. In our analyses we tried to identify similar interventions using social media, however these multimodal and information rich approaches have not been implemented, or broadly explored, in the context of social media interventions.

In our selected cases, the precision component tailored the intervention to the participants. All participants received the same type of intervention, along with the same social media functionalities. Individual interventions involved tailoring the calorie intake and exercise programs used in Case 2 [ 20 , 23 , 24 ] to more stratified interventions as in Case 3 [ 19 , 25 ] where the intervention is “culturally” tailored.

### 4.2. Personalization factors and behavior components in social media precision prevention interventions

The most frequently employed customized strategies involved the use of persuasion and incentivization. This finding is expected, considering that these approaches are firmly established components of interventions [ 26 , 27 ]. When employed by those individuals with whom one trusts, such as a physician, persuasion can effectively motivate people to change their diet, become more active, or undertake other significant lifestyle changes. In this analysis, the use of evidence-based messaging supported efforts to moderate alcohol consumption, reduce weight after giving birth, and increase physical activity among women with hypertension. Investigators delivered persuasion via personal engagement with individuals, which is associated with increased success in behavior change [ 28 ]. The incorporation of personal support for behavior change, present in two of the cases, is another factor that boosts success [ 29 ] and can thus make social media-based interventions a relevant, useful approach.

Incentivization has been shown to motivate some individuals to complete desired behavior change tasks, such as adopting regular physical activity or managing dietary intake. In this analysis, it supported mitigation of alcohol consumption, weight-loss goal setting and achievement, and reduction of high-risk behaviors such as excessive drinking. Even relatively small incentives such as payments of US$20 were effective in influencing the participants' actions. The reach of social media into a broad range of diverse populations [ 1 2 3 4 ] offers the possibility of implementing incentivization-based interventions in communities that may be difficult to reach via clinic-based initiatives.

Although education is a highly effective component for behavior change [ 16 ], it was not a primary strategy for behavior change efforts among our selected cases. Coercion, or creating expectation of punishment, played a minimal role in these behavior change interventions, which is not surprising given health care providers' recognition of trust as a key component of the patient-provider relationship.

Training and modeling are behavior change components known for their effectiveness [ 16 ], but were not used in the cases evaluated in this work. It may be that social media platforms offer limited options for use of content delivered solely via digital platforms, or that other aspects of the intervention (e.g., duration, timing) made it difficult to incorporate these components. Participant characteristics such as preferred language also may have played a role in the selection of strategies.

As previously discussed, an aspect of precision prevention is the possibility of using multimodal datasets to tailor the prevention. In this regard, social media can be considered as a relevant tool for digital phenotyping and therefore their use can be used as a relevant source of information for the development of precision prevention [ 30 ]. There is an increasing body of literature advocating for the use of social media as part of the digital phenotyping efforts [ 31 32 33 ], however the use of this source of data does have some ethical challenges related with the magnitude of the information collected, privacy, and General Data Protection Regulation compliance.

### 4.3. Limitations

Our paper has certain limitations that warrant acknowledgment. Our search for examples of research studies focused exclusively on a single source. The cases presented in this paper are intended solely as illustrative examples. While they offer valuable insights, they may not encapsulate the entirety of the diverse landscape of precision prevention interventions employing social media. It is plausible that other studies, not captured in our selection, might more accurately represent the concept of precision prevention.

## 5. Conclusion

In conclusion, social media has the potential to contribute to the advancement of precision prevention. Using the Behavior Change Wheel (BCW) framework, it was found that persuasion and incentivization were the most commonly used components in personalized interventions in the four chosen cases. Education was provided in the form of posts or website links to participants. Coercion played a minimal role in the interventions; however, this can be expected as health care providers strive to build a trusting relationship with a patient. The other BCW intervention components of training, restrictions, and modeling were not used in any of the chosen studies, which may be due to limitations in the chosen social media platform, or to the personal characteristics of the participants themselves. In this review, we only aimed to analyze examples of what can be considered precision prevention interventions delivered through social media. Thus, additional research is needed to further explore the personalization elements and the behavioral components employed in this type of interventions. Further studies are also needed to determine if the lifestyle changes last, and if the needed safeguards put in place to protect the privacy and security of personal health information of participants is sufficient.
